# The effect of different concentrations of gold nanoparticles on growth performance, toxicopathological and immunological parameters of broiler chickens

**DOI:** 10.1042/BSR20194296

**Published:** 2020-03-27

**Authors:** Eman I. Hassanen, Eman A. Morsy, Ahmed M. Hussien, Marwa A. Ibrahim, Khaled Y. Farroh

**Affiliations:** 1 Department of Pathology, Veterinary Medicine, Cairo University, Giza, Egypt; 2 Department of Poultry Diseases, Veterinary Medicine, Cairo University, Giza, Egypt; 3 Department of Toxicology and Forensic Medicine, Veterinary Medicine, Cairo University, Egypt; 4 Department of Biochemistry and Molecular Biology, Veterinary Medicine, Cairo University, Giza, Egypt; 5 Department of Nanotechnology, Agricultural Research Center, Giza, Egypt

**Keywords:** gene expression, gold nanoparticles, histopathology, inflammation, performance

## Abstract

The present study aimed to evaluate what dosage of gold nanoparticles (GNPs) would improve growth performance, antioxidant levels and immune defense in broiler chickens. The experiment was carried out on 90 one-day-old mixbred Cobb chicks. The birds were allocated into three groups with three replicates. Group (1) kept as a negative control. Groups (2) and (3) received 5, 15 ppm GNPs via drinking water weekly for 35 days of chicks’ life. Blood samples were collected at 8, 15, 22 and 36 days for oxidative stress evaluations and immunological studies. The birds were slaughtered at the ages of 36 days and thymus, spleen, busa of Fabricius and liver were collected for histopathological description, RT-PCR analysis and DNA fragmentation assay. Our results confirmed that adding of 15ppm GNPs in drinking water were induced remarkable blood oxidative stress damage, histopathological alterations, up-regulation of IL-6, Nrf2 gene expression, and DNA fragmentation in the examined immune organs of the broiler chickens as well as a significant reduction in the antibody titer against Newcastle (ND) and avian influenza (AI) viruses were noticed. On the other hand, the group received 5 ppm GNPs noticed better growth performance with the enhancement of the final food conversion ratio (FCR) without any significant difference in the previous toxicological and immunological parameters compared with the control groups. We suggest that feeding of 5ppm GNPs could improve the antioxidant capacity, immunity and performance in poultry but further food quality assurance tests are required in the future to confirm its safety for people.

## Introduction

Nanotechnology is defined as the comprehension and control of matter at the nano scale, at measurements between roughly 1 and 100 nanometers [1,2]. In the ongoing years, nanotechnology had been advance and had the most impact on all parts of human, animal, environmental and industrial life [3]. Several nanoparticles were incorporated into foods/feeds like those engineered to provide encapsulation systems, as micelles, liposomes, delivery of food/feed ingredients, and those customized for use in food/feed packaging such as biosensors, identification markers, and antimicrobials [4]. The utilization of nanotechnology is extremely fluctuated. In the field of veterinary medicine and animal production, there is a developing enthusiasm for the utilization of nanotechnology that can be utilized as a supplemental source of trace minerals (Na_2_O, MgO, Al_2_O_3_, SiO_2_, K_2_O, CaO, TiO_2_ and Fe_2_O_3_) in diets [5]. Nanotechnology used extensively in poultry and animal farms as well as in milk, eggs and meat industries to improve the quality and quantity of the live stock products [6]. It can also reduce the time required for meat and eggs production through improving animal’s reproduction, and food conversion ratio. Nanotechnology can increase the average daily weight gain of broiler chickens, and lower feed conversion [7]. The importance of nanotechnology in broiler farms and chicken meat productions can’t be completely valued yet due to loss of sufficient knowledge and data in this area. From another perspective, addition of low levels of certain antibiotics either in food or in drinking water is a typical practice in poultry breeding and provides financial advantages by increasing weight gain and improved feed efficiency [8]. On the other hand, recently published data reported that adding antibiotics in feed has been prohibited because of the potential development of antibiotic-resistant human pathogenic bacteria and their residues in poultry production [9–11].

Gold and its compounds have long been utilized as medicinal agents throughout the history of civilization [12]. Gold nanoparticles (GNPs) have an immense application in nano medicine, and nano biotechnology field because of their noncytotoxic, large surface area and biocompatible properties [13]. The dose-dependent toxicity of GNPs is seldom talked about uniquely in poultry field. The toxicological effects of GNPs are close related to their shape, size, concentrations, exposure time, capping agent, surface chemistry, animal species and route of administration [14,15]. GNPs can reach to the gastrointestinal tract (GIT) through feeding, watering or from the administration of therapeutic nano-drugs. Uptake of GNPs in the GIT relies up on the mucociliary and cellular reactions of the GIT. The smaller particle diameter, the faster is the diffusion through GIT mucus to reach the cells of the intestinal lining, and reach to the blood circulations [16,17]. Following uptake from the GIT, NPs at 100 nm or less can translocate through lymphatic and reach to the liver and spleen [18]. Smaller NPs (50 nm or less) are capable of being taken up by the villus epithelium [19] and directly enter the blood stream, then predominantly scavenged by the liver and spleen. Previous study reported that the *in ovo* supplementation of GNPs conjugated with taurine to the chicken embryo had the ability to enhance the breast muscle quality and quantity *via* activating molecular mechanisms i.e. PCNA, VEGF [20].

In any case, nanotechnology is likewise a 2-fold edged sword; in spite of the fact that nanotechnology can be utilized in numerous territories and numerous beneficial impacts have been achieved, it also has numerous adverse effects to humans, animals, plants, and the environment. Government and regulatory authorities; environmental, health and safety councils; and scientific authorities everywhere throughout the world are understanding the significance of nanotechnologies hazard assessment [21]. In general, the mechanism of NPs cytotoxicity based on several ways such as oxidative stress damage, apoptosis, DNA damage and genotoxicity [22]. The dose-dependent effect of NPs were examined by Hao et al. [23] who reported that addition of 100 and 200 mg/l TiO2-NPs in water showed dose-dependent oxidative stress manifested by a significant decrease in antioxidants levels as superoxide dismutase (SOD), catalase (CAT), and peroxidase (POD) activities associated with significant increase in lipid peroxidation (LPO) levels in different fish tissues. Another study showed that low concentrations of GNPs (0.13 ppm) did not cause an obvious decrease in body weight or appreciable toxicity in mice. On the other hand, high concentrations of GNPs (2.2 ppm) induced decreases in body weight associated with remarkable toxicity [24]. Other *in vitro* study described the cytotoxicity of GNPs on human dermal fibroblasts in the form of apoptosis based on size, concentration and exposure time [25].

GNPs caused a significant generation of reactive oxygen species (ROS) and up-regulation of genes involved in cellular stress and toxicity. Among these, the transcription factor nuclear factor erythroid 2 (NFE2)-related factor 2 (Nrf2) that considered as a main regulator of cell survival via induction of phase II detoxification and antioxidant defense enzymes [26]. Induction requires a common DNA sequence called antioxidant response element (ARE) that resembles the NFE2-binding motif [27]. ARE is commonly found in the promoter region of genes encoding phase II detoxification and antioxidant enzymes [28]. Nrf2 is the most powerful inducer of ARE-mediated expression among these transcription factors [29]. Additionally, it had the ability to mediate induction of several drug-metabolizing enzymes including detoxifying enzymes NAD(P) H:quinone oxidoreductases (NQO1 and NQO2), the glutathione (GSH) *S*-transferase, γ-glutamylcysteine synthetase, and heme oxygenase (HO)-1 [30]. Several studies showed positive correlation in between Nrf2 and IL-6 gene expressions [31]. IL-6 plays a key role in the process of inflammation, is able to induce fibrinogen, serum amyloid A protein, the acute phase response, and is one of the most important mediators of fever.

The systemic toxicity of the GNPs was linked to major organ damage such as liver, spleen and lungs of mice as well as it is associated with marked elevation of pro-inflammatory cytokines as IL-1, IL-6 and TNF [32].

In agriculture and animal production fields, a particular group of NPs have been counted as substitutes for growth-promoting antibiotics by modifying the tissue accumulation and the bacterial resistance profiles in animal’s nutrition [33].

Nanotechnology provides potential enthusiasm for numerous fields and applications, especially the biomedical sciences, nano medicine and veterinary medicine. The application of gold nanoparticles was examined extensively in human medical field and further studies were attempted in rodents broadly. Most of these studies mentioned the toxicity of GNPs based on size, surface charge and capping agents but little known about their toxicity based on the concentration and dose differences. The use of GNPs in the veterinary medicine and poultry industry field is relatively innovative. So, this is the first study aimed to investigate the effect of different concentrations of GNPs on growth performance, oxidative stress parameters, immune status, DNA fragmentation, proinflammatory cytokine levels and histopathological descriptions of different organs of broiler chickens. From our study, we can pick the best concentration of GNPs that might be utilized for improvement of growth performance and immunological defense of broiler chickens.

## Materials and methods

### Preparation and characterization of gold nanoparticles

Gold nanoparticles colloidal solution (25 ± 5 nm) was synthesized by chemical reduction method through the reduction of gold (III) chloride hydrate (99.995% HAuCl_4_, Sigma-Aldrich, St. Louis, MO, U.S.A.) with Tri-sodium citrate dehydrate (99%, Sigma-Aldrich, St. Louis, MO, U.S.A.) under boiling conditions [34]. Briefly, 50 ml (0.03 mM) HAuCl_4_ was brought in the beaker to boil under stirring for 5 min. Then, 0.5 ml Tri-sodium citrate dehydrate (1%) solution was added at once under continuous stirring. The solution color turned bright red forming gold nanoparticles colloid, then left to cool and proceeds for physiochemical characterization.

The chemical structure of the prepared GNPs has assessed using X-ray Diffraction (XRD) technique. The corresponding XRD pattern was recorded in the scanning mode (X'pert PRO, PAN analytical, Netherlands) at 40 kV, 30 mA and interpreted by the standard ICCD library installed in PDF4 software. Dynamic light scattering (DLS) measurement of the size and zeta potential was undertaken using a Nano-zeta sizer (Malvern, ZS Nano, U.K.). The morphology of GNPs was imaged by High-Resolution Transmission Electron Microscope (HR-TEM) operating at an accelerating voltage of 200 kV (Tecnai G2, FEI, Netherlands).

### Birds and experimental design

Ninety one-day-old Cobb mixed broiler chicks were obtained from El-Hwamdya, Giza, Egypt. The birds were weighed and randomly allocated into three treatment groups with three replicates of 10 birds each so that their initial body weights were similar across all the groups. Chicks were raised in deep letter system with straw bedding and the housing was controlled at standard conditions of temperature, humidity and ventilation and maintained on a 24-h constant-light regime throughout the trial period. The birds received water and food *ad libitum*. As well balanced ration (including starter and grower ration) without any additives was used for the rearing period of broiler chickens. All birds received the following vaccines: Live Newcastle (ND) and IBV(HitchnerIB) was administered by ocular route at 7th day of age, avian influenza reasserting inactivated vaccine (H5N1) was administered by s\c at 10th day of age, live intermediate vaccine against IBD virus strain winter field was administered by ocular route at 12th day of age, and LaSota vaccine was also administered by ocular route at 18th day of age.

The birds were divided as follow: Group (1) kept as the control without any treatments. Groups (2) and (3) received 5, and 15 ppm gold nanoparticles (GNPs) via drinking water respectively, weekly at 1, 7, 14, 21, 28 and 35 days of chicks’ life.

Doses of GNPs selected according to previous study [35]. We selected the lowest (5 ppm) and medium (15 ppm) concentrations of GNPs to achieve the best results, highest growth performance and immune status with minimum toxicity, with minimum financial cost.

### Sampling

Blood samples were collected from the wing vein of 3 chicks per replicate at 8, 15, 22 and 36 days. Centrifugation performed at 4500 rpm for 5 min to get clear serum samples which preserved at –20°C till used for immunological study and oxidative stress evaluations. The birds were slaughtered by exsanguination at the ages of 36 days then the immune organs such as liver, spleen, thymus, bursa of Fabricius were collected and preserved in 10% neutral buffered formalin for histopathological examinations. Some tissue specimens from the liver and bursa of Fabricius were preserved at –80°C for RT-PCR analysis and DNA fragmentation assay.

### DNA laddering assay

DNA laddering assay was conducted in liver tissue because it is the most edible organ in chickens and it is the site for drugs detoxification. Briefly, the tissues were homogenized in lysis buffer (10 mM Tris-HCl, pH 7.4, 10 mM EDTA, 0.5% Triton x100) then centrifuged at 13800 *xg* for 15 min. The pellets containing total intact DNA (P) and the supernatants containing smaller fragments of DNA (S). Both the S and P fractions were treated with 0.5 ml of 10% trichloroacetic acid (TCA) and were left overnight at 4°C. An 80 μl of 5% TCA was added and incubated at 90°C for 15 min. Freshly prepared 1 ml diphenylamine reagent (1.5 g of diphenylamine dissolved in 100 ml acetic acid, 1.5 ml of conc. sulfuric acid and 0.50 ml of acetaldehyde (16 mg/ml)) was added in each sample, tubes were allowed to stand overnight at room temperature and OD was recorded at 600 nm [36]. DNA laddering percentage was calculated as: % DNA laddering [*S*/(*S* + *P*)] × 100.

### Agarose gel electrophoresis of the fragmented DNA

The agarose gel electrophoresis was done for the DNA fragments extracted from the supernatant portion using DNeasy kit (Qiagen) electrophoresed in 1.5% agarose gel for 90 min at 5 V/cm and visualized with ethidium bromide.

### Quantitative real-time PCR for Nrf-2 and IL-6 genes

Total RNA was isolated from bursa of Fabricius tissue using RNeasy mini kit (Qiagen) according to the manufacturer’s instructions. First-strand cDNA was generated by reverse transcription of 10 μg RNA samples [37]. The primer set used for Nrf-2 are Forward primer: ACGCTTTCTTCAGGGGTAGC; Reverse primer: GTTCGGTGCAGAAGAGGTGA and those of the IL-6 were Forward primer: CTGCAGGACGAGATGTGCAA; Reverse primer: AGGTCTGAAAGGCGAACAGG. Real-time PCR was done using a Real-Time PCR System (Applied Biosystems, U.S.A.) which was run for 40 cycles of denaturation at 95°C for 45 s, annealing at 59°C for both genes for 45 s and extension at 72°C for 45 s. The sizes of all amplicon (170bp for Nrf-2 and 175bp for IL-6) were confirmed by 2% agarose gel electrophoresis stained with SYBR Safe DNA gel stain (Invitrogen) [38].The β-actin gene was amplified in the same reaction to serve as the internal control [39]. Each assay was repeated three times, and the values were used to calculate the gene/ β-actin ratio, with a value of 1.0 used as the control (calibrator). The normalized expression ratio was calculated using the Mxpro software [40].

### Immunological parameters (HI)

To decide the impact of various concentrations of GNPs on humeral immunity, anti-ND and anti-AI vaccine antibody titers were assessed in serum samples at 8, 15 and 22 days old using hem agglutination inhibition (HI) test. Briefly, dilutions of serum are incubated with virus, and erythrocytes are added. After incubation, the HI titer is read as the highest dilution of serum that inhibits Hemagglutination [41].

### Oxidative stress evaluations

Serum samples collected at 36 days old were evaluated for lipid peroxidation, expressed by malondialdehyde (MDA) formation, following the method described by Ohkawa et al*.* [42], and Catalase (CAT) activity was measured as the method described by Aebi [43], using commercial kits (Biodiagnostics, Cairo, Egypt).

### Growth performance assessment:

The body weight of 10 birds in each group was weekly estimated as well as feed intake per group were likewise estimated weekly on the same days of birds weighting to figure the food conversion ratio (FCR, g feed/ g bwt gain) that determined by the proportion of feed intake to weight gain per bird\week according to Timmerman et al [44].

### Histopathology and histomorphometric analysis of the immune organs

Tissue specimens from the liver, spleen, thymus and bursa of Fabricius were gathered from all birds at 36 days old. The specimens were fixed in 10% neutral buffer formalin (pH 7.0) then processed by conventional methods and sliced at 4 μm to acquire paraffin-embedded tissue sections stained by hematoxylin and eosin (H&E) for histopathological examinations [45].

Microscopic grading and scoring of the hepatic sections were done to assess the level of seriousness of the noticed histopathological alterations as indicated in the strategy depicted by Hassanen et al [46]. At least, 7 hepatic tissue sections representing 7 birds per group in each replicate were utilized for evaluating the following pathological parameters: hepatocellular cytoplasmic vacuolization, necrosis, vascular congestion and hemorrhage. All of the above parameters surveyed and scored as slight, mild, moderate and severe pathological alterations, as to pursue (0) normal histology; (1) slight <25%; (2) mild 25–50%; (3) moderate 50–75%; (4) severe >75% of the tissues affected. The severity of lesions of the lymphoid organs (bursa, thymus, spleen) was scored by using a scale of scoring system numbered from 0 to 5, represented the degree of lymphocytic depletion and lymphocytolysis, corresponding to normal, <25%, 25:49, 50:69, 70:90, >90% of the tissues affected respectively [47].

Concerning bursa, spleen and thymus, five sections relating to five birds for every gathering were analyzed using ImageJ software. The mean percentage area of the cortical bursal follicles was decided in 12 complete follicles as the strategy portrayed by Muniz et al. [48]. The percentage of the follicular cortex was measured as a total follicular area less medullar area in each follicle. The follicular lymphoid cell percentage areas of the spleen were additionally decided by the technique portrayed by Wilson [49]. The percent area of the follicular lymphoid cell was determined with respect to the total area of interest. Cortical: medullar ratio in the thymus gland was likewise decided.

### Statistical analysis

Statistical analysis was performed with SPSS version 16.0 software (SPSS Inc., Chicago, IL, U.S.A.). Data were expressed as means ± SE. Comparison of means was performed by one-way analysis of variance (ANOVA) followed by Student’s *t*-test. A value of *P* ≤ 0.05 was considered statistically significant.

## Results

### Characterization of GNPs

**Figure 1:**
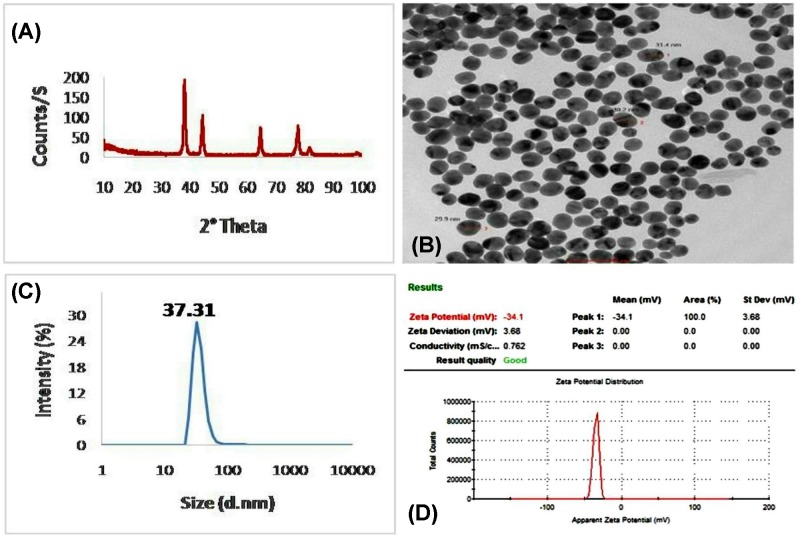
Characterization of gold nanoparticles (GNPs) (**A**) XRD pattern analysis indicating the formation of GNPs with cubic unit crystal. (**B**) HR-TEM image showing spherical shaped GNPs with average size 30.5 nm.(**C**) Particle size distribution of the prepared GNPs showing the average size of 37.31 nm. (**D**) Zeta potential of the prepared GNP was −34.1 mV.

The physicochemical features of the GNPs were illustrated in Figure 1. The XRD results of the GNPs phase formation, based on Bragg’s reflections law, were showing in Figure 1A. Characteristic diffraction pattern showed narrow peaks and sharp intense at 38.2°, 44.4°, 64.6°, 77.6° and 81.7° 2θ angles that are corresponding to *hkl* parameters of (111), (200), (220), (311) and (222), respectively. The diffraction pattern was compared with the standard ICCD library installed in PDF4 software, card no: (04-007-8000). The HR-TEM electrograph, used for determination of the nanoparticle morphology and size, revealed well-uniformed spheres with an average size of 30.5 nm (Figure 1B). The particle size distribution curve obtained from DLS measurementswas 37.31 nm (Figure 1C). The measurement of the GNPs surface charge and zeta potential was −34.1 mV by using DLS technique (Figure 1D).

### DNA fragmentation and m-RNA levels of IL-6 and Nrf-2

**Figure 2:**
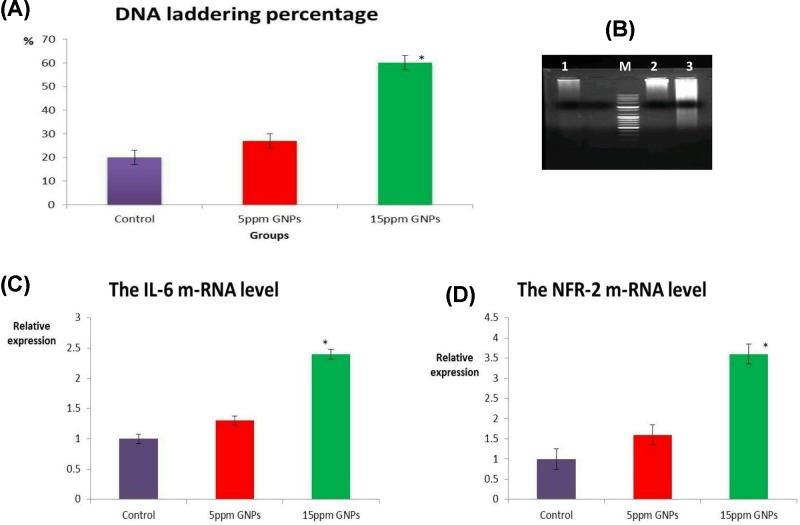
DNA percentage and m-RNA levels (**A**) Bar chart of DNA laddering percentage in different groups. (**B**) Electrophoresis of the DNA fragments on 1.5% agarose gel. M: 100 bp DNA ladder; lane 1: control group; lane 2: 15 ppm GNPs; lane 3: 5 ppm GNPs. (**C**) Bar chart of IL-6 mRNA levels, and NFR-2 mRNA levels (**D**) in different groups. Values were presented as mean ± SEM (*n*=7 birds/ group). * means significant different from the control group at *P*≤0.05.

The highest DNA laddering percentage was detected in the hepatic tissue of group received 15 ppm GNPs (60%) followed by those received 5 ppm GNPs (27%) as illustrated in Figure 2A. The same results were confirmed by the electrophoretic mobility of the DNA fragments on the agarose gel. The DNA laddering was clear and prominent in the liver of the group received 15 ppm GNPs (Figure 2B).

### Quantitative real-time PCR for Nrf-2 and IL-6 genes

Up-regulation of Nrf-2 and IL-6 mRNA levels was reported in the bursal tissue of group received 15 ppm GNPs compared with control negative as shown in Figure 2C,D. There was no significant difference in Nrf-2 and IL-6 mRNA levels between the control group and those received 5 ppm GNPs.

### The effect of GNPs on antibody titer and oxidative stress parameters

**Figure 3:**
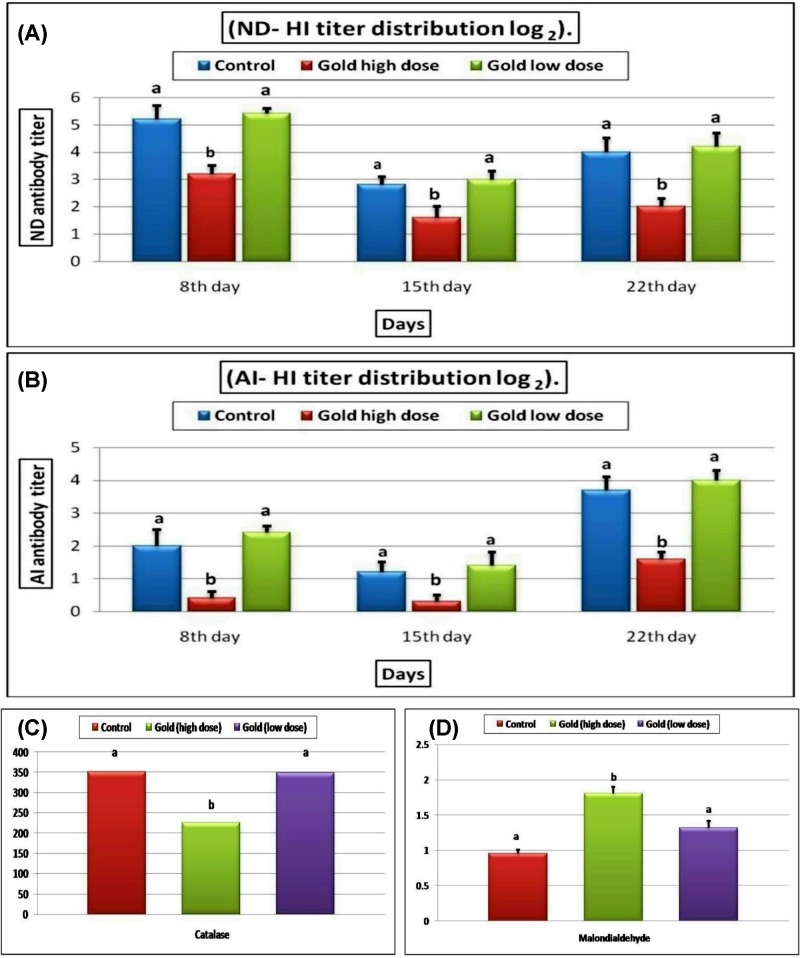
Blood antibody titer and oxidative stress parameters Bar chart representing the effect of different concentrations of GNPs on blood antibody titer against ND (**A**) and AI virus (**B**), serum MDA level (**C**), Blood CAT activity (**D**) in different groups of broiler chickens. All values were presented as mean ± SEM (*n*=7 birds/ group). Values with different letters were significantly different at *P*≤0.05.

At 8 days old, antibody titers against ND and AI in all groups were quite similar to each other. At 15 and 22 days old, there was a significant decrease in the antibody titer against both ND and AI virus in the group received 15 ppm GNPs, but not in the group received 5 ppm GNPs, compared with the control group (Figure 3A,B)**.** The impact of various concentrations of GNPs on serum MDA level and CAT activity was graphically illustrated in Figure 3C,D**.** The results revealed that birds received 15 ppm GNPs, but not 5 ppm GNPs, showed a significant increase in the serum MDA levels and a significant decrease in blood CAT activity compared with the control group.

### Histopathological description of the immune organs

**Figure 4:**
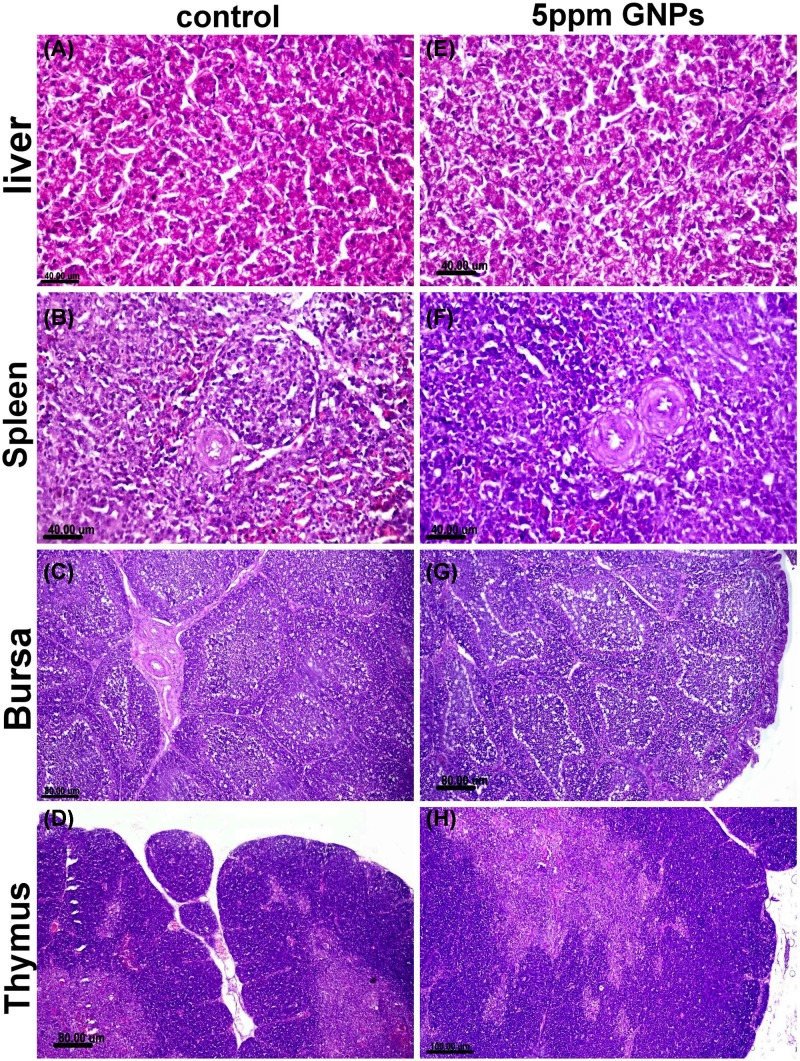
Histopathological examination of different organs from groups recieved 5 ppm GNPs Photomicrograph of liver, spleen, bursa of fabricius and thymus tissue sections stained with (**H,E**) of control negative group (**A–D**) and those receiving 5 ppm GNPs (**E–H**) showed normal histology with minimum pathological alteration in group receiving 5 ppm GNPs.

All the examined immune organs (liver, spleen, thymus and bursa of Fabricius) in the control group showed normal histology. Birds received 5 ppm GNPs noticed mild cytoplasmic hepatocellular vacuolization within hepatic sections, mild lymphoid cell depletion with lymphocytosis in both splenic and bursa lymphoid follicles. However, spleen tissue sections showed normal histology as illustrated in Figure 4.

As opposed to control gathering, birds received 15 ppm GNPs exhibited severe histopathological alterations in all the examined organs.

**Figure 5:**
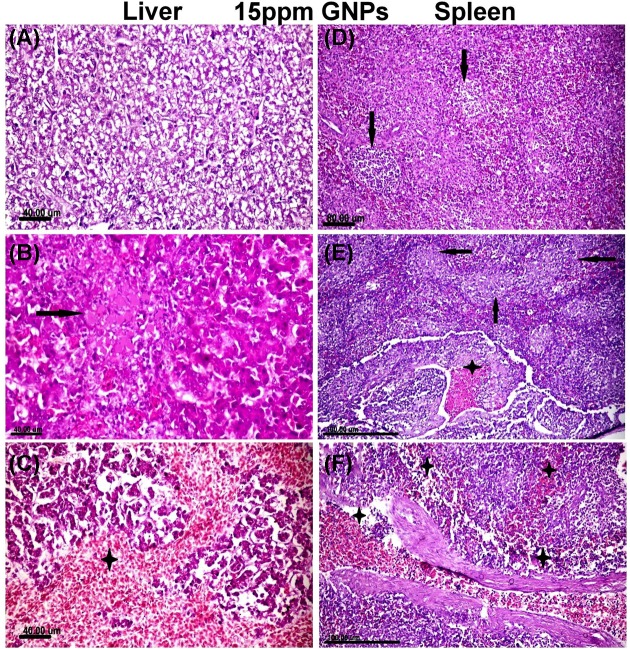
Histopathological examination of different organs from groups recieved 15 ppm GNPs (**A–C**) Photomicrograph of liver tissue sections of group received 15 ppm GNPs showed. (A) Diffuse hepatocellular cytoplasmic vacuolization with distortion of the hepatic architecture. (B) Focal irregular region of hepatocellular coagulative necrosis (arrow) containing hepatocytes with eosinophilic cytoplasm and pyknotic or completely lysed nuclei. (C) Large area of hepatocellular hemorrhage (star) with necrosis of the surrounding hepatocytes.(**D–F**) Spleen tissue sections of group received 15 ppm GNPs showed. (D) Lymphoid cell depletion with necrosis (arrows) in most of lymphoid follicles. (E) Congestion in blood vessels (star) with multifocal necrotic areas replacing the white pulp (arrows). (F) Severe congestion of the spleenic cord and sinuses (star). All figures stained with H&E.

Liver tissue sections noticed random multifocal regions of coagulative necrosis that invaded by mononuclear inflammatory cells throughout the hepatic parenchyma (Figure 5A). The majority of the hepatocytes showed vacuolar degeneration and fatty changes (Figure 5B). Enormous focal territories of hemorrhage were recorded within the hepatic parenchyma especially in the sub capsular area (Figure 5C).

Spleen tissue sections noticed moderate lymphoid cell depletion and lymphocytolysis in most of the lymphoid follicles associated with reticular cell hyperplasia (Figure 5D). Multifocal areas of necrosis replacing the majority of the white pulp were recorded (Figure 5E). Red pulp and splenic sinuses were congested and filled with fibrin and hemosiderin pigments (Figure 5F). Perisplenitis was observed and characterized by thickening of the splenic capsule by edematous exudates, hemorrhage and minimum inflammatory cells infiltrations.

**Figure 6:**
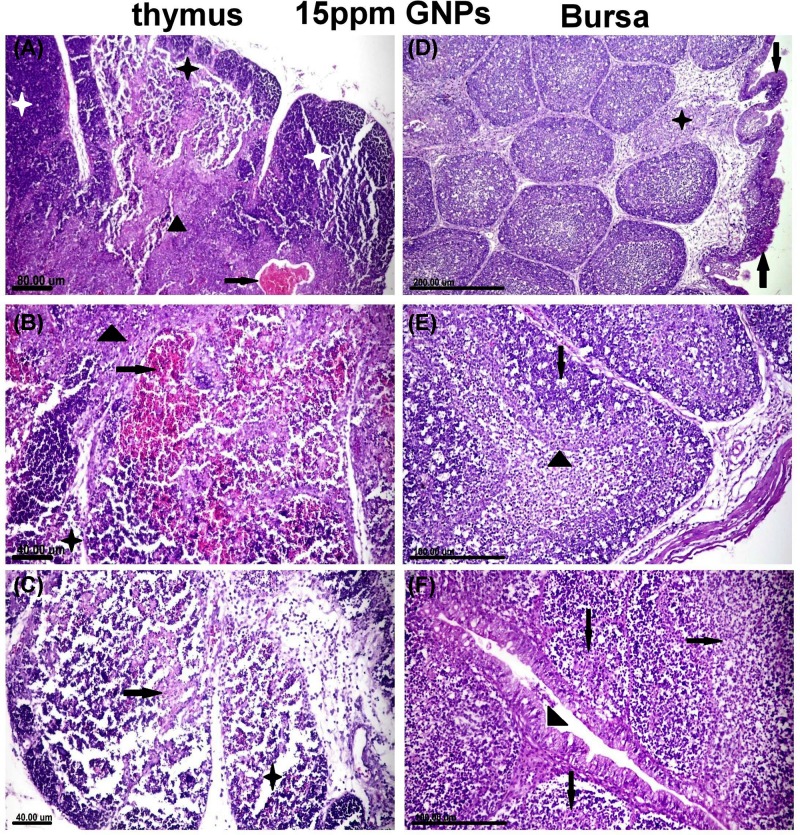
Histopathological examination of different organs from groups recieved 15 ppm GNPs (**A–C**) Photomicrograph of thymic tissue sections of group received 15 ppm GNPs showed. (A) Thinning of cortical width associated with lymphoid cell depletion and reticular cell hyperplasia in thymic cortex (star) and medulla (arrow head), congestion of medullar zone was noticed (arrow). (B) Sever lymphoid cell atrophy and reticular cell hyperplasiain both cortex (star) and medulla (arrow head) associated with medullar hemorrhage (arrow). (C) Extensive lymphoid cell depletion (star) with prominent reticular cells (arrow) in thymic cortex. (**D–F**) Bursa of fabricius tissue sections of group received 15 ppm GNPs showed. (D) Atrophy of bursal lymphoid follicles associated with widening in bursal septa with edematous exudates and inflammatory cells (star) with irregular folding of surface epithelium (arrows). (E) Lymphoid cell depletion and extensive lymphocytolysis (arrow) in both cortex and medulla of bursa follicles associated with prominent basement membrane (arrow head) in between. (F) Severe destruction and necrosis of bursal follicles with necrotic cell debris in its lumen (arrows), surface epithelium noticed hyperplasia of goblet cells (arrow head). All figures stained with H&E.

Thymus noticed extensive cortical necrosis (Figure 6A) accompanied by medullar congestion and hemorrhage in the majority of the lobules (Figure 6B). Thinning in the cortical layer was observed and associated with lymphocytolysis and presence of a high number of cells surrounded with hollow zone represented apoptotic or necrotic lymphocytes. Extensive lymphoid cell atrophy and reticular cell hyperplasia were demonstrated in both thymic cortex and medulla (Figure 6C).

Bursa of fabricius showed lymphoid cell atrophy of bursa follicles associated with irregular folding of the surface epithelium. Expansion of interfollicular septa by edematous exudates and inflammatory cells infiltrations were observed in most sections (Figure 6D). There were extensive necrosis and lymphocytic cell depletion in the majority of bursal follicles with prominent basement membrane between follicular cortex and medulla associated with a massive number of apoptotic lymphocytes (Figure 6E). Some sections showed severe destruction of the bursal follicles with intraluminal necrotic cell debris (Figure 6F).

**Figure 7:**
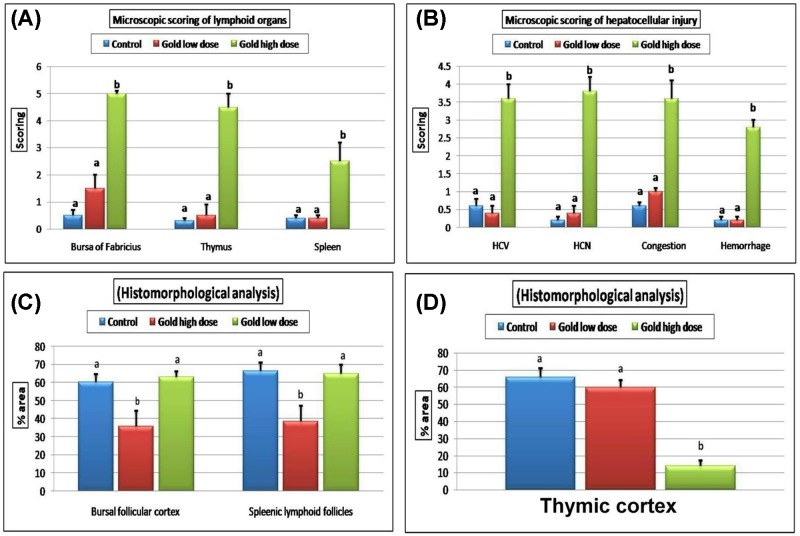
Microscopic lesion scoring and morphometric analysis of different organs Bar charts representing (**A**) microscopic scoring of the lymphoid organs (spleen, bursa of fabricius and thymus). (**B**) Microscopic scoring of the hepatocellular injury. (**C**) Mean percentage area of bursal follicular cortex and spleenic lymphoid follicles. (**D**) Mean percentage area of thymic cortex in different groups. Values were presented as mean ± SEM (*n*=7 birds/ group in each replicates). Values with different letters within the same row were significantly different at *P*≤0.05; HCV, hepatocellular cytoplasmic vacuolization; HCN, hepatocellular necrosis; CV, central vein; PA, portal area.

Microscopic scoring and morphometric analysis showed a significant increase in the microscopic score in all of the examined organs in the group received 15 ppm GNPs compared with the control group (Figure 7A,B). The morphometric analysis showed a significant decrease in the mean % area of the bursa cortical layer, splenic lymphoid follicles (Figure 7C) and thymic cortex (Figure 7D) in the group received 15 ppm GNPs compared with the control group.

### The effect of GNPs on growth performance

**Figure 8:**
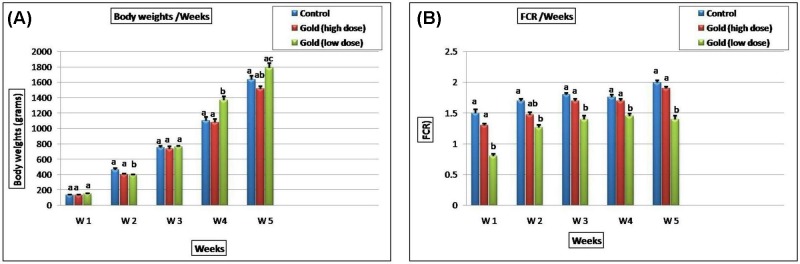
The effect of GNPs concentrations on the growth performance Bar charts representing (**A**) mean body weight (gm/wks). (**B**) Feed conversion ratio (FCR) in different groups. Values were presented as mean ± SEM (*n*=7 birds/ group in each replicates). Values with different letters within the same row were significantly different at *P*≤0.05.

The impact of various concentrations of GNPs on growth performance is illustrated in Figure 8. A significant increase in the average body weight (ABW) and feed conversion ratio (FCR) was observed in the group received 5 ppm GNPs, but not in the group received 15 ppm GNPs, compared with control group all over the experimental period.

## Discussion

Nanotechnology will play an indispensable role in the future in area of animal and poultry nutrition research [50]. Nanoparticles have many advantages; for instance, it can be delivered through the stomach wall into body cells more quickly than the larger particle size. Besides, nano-additives can also be incorporated in micelles or capsules of protein or another natural feed ingredient. Although wide applications of GNPs in medicine as an anti-oxidant, anti-inflammatory, anti-angiogenic and anti-carcinogenic material [51,52], their uses in veterinary medicine still unclear especially in poultry field. So, the present study was considered to be the first study, according to previous literature, designed to investigate the effect of two different doses of GNPs on growth performance, immunological parameters, oxidative stress damage, pro-inflammatory cytokines, DNA fragmentation assay and histopathological organizations in some organs such as liver, spleen, thymus, bursa of fabricius in broiler chickens.

Data of the present study indicated that adding of 5 ppm GNPs in the drinking water weekly for 5 days significantly improved LBW, BWG and FCR of broiler chickens all over the experimental period while group of birds that received 15 ppm showed mild improvements in LBW, BWG and FCR at last 2 weeks compared with control group. We suggest that the favorable effects of using GNPs as a growth promoter may be related to several factors like concentration, route of administration, dose, size and nature of nanoparticles. In other words, Desai et al. [53] observed that the oral uptake of nanoparticles increases surface area, which in turn could increase the absorption and utilization of minerals required for improving the growth performance of broiler chickens. Likewise, Weiss et al. [54] recorded that the ingredients’ nanoparticle size might increase the functionally or bioavailability of ingredients and nutrients, leading to increased BW and BWG. In addition, the increase in LBW and BWG of broiler chickens may be attributed to the fact that nanoparticles have a surface area much larger than micro-particles. Hence, we assume that as the size of nanoparticles decreases, the surface area for chemical reactions increases, leading to better digestion and utilization of minerals in the GIT [55]. A parallel study was conducted by Mushtaq et al. [56] who reported that intraperitoneal injection of GNPs in mice improved the absorption of amino acids and glucose that are essential for body growth. This finding may explain the significant improvement in the growth performance of broiler chickens obtained from the present study. Moreover, the interest in using nanotechnology as a new advanced tool in broiler nutrition indicates that nano-scale particles have chemical and physical properties that completely differ from those of large-scale particles [57].

Histopathological findings of the entire examined organs showed normal histology or mild pathological lesions in 5 ppm GNPs received group suggesting the healthy status of broiler. On the other hand, the group received 15 ppm GNPs showed severe histopathological alterations in all the examined organs. Concerning immune organ, lymphoid cell depletion and lymphocytolysis in the lymphoid follicles of the bursa of Fabricius, spleen, and thymus gland were considered as the most observable lesions in this group. Our finding indicates the immunosuppressive effect of 15 ppm GNPs on broiler chickens that may be correlated to depletion of T- and B-lymphocytes. Additionally, our results showed also a significant reduction in antibody titer against AI and ND with a significant up-regulation of IL-6 mRNA levels in the group received 15 ppm GNPs. It is well known that the phagocyte and lymphocyte constitute the first barrier to nanoparticle penetration of animal tissues and cells. Therefore, the study of GNPs interactions with the phagocyte cells, the mechanisms of intracellular uptake, and the responses of immune cells to GNPs is undoubted of major interest. Further, several studies on the *in vivo* GNPs effect confirmed that GNPs induced size and dose-dependent effects on the eukaryotic cells. Małaczewska [58] demonstrated that mice, after being orally administered with GNPs showed increased the phagocytes activity and the percentage of both B and CD4+/CD8+ double-positive T cells. On the other hand, the effect of the highest dose can be considered pro-inflammatory and immunotoxic, because of the stimulated pro-inflammatory cytokine as TNF, IL-2 and IL-6 synthesis was accompanied by a drastic decline in the proliferative activity of lymphocytes and extensive pathological lymphocytolysis and lymphocytic depletion in different lymphoid organs [59]. Several recent publications have been reported the dose-dependent GNPs induced changes (both increasing and decreasing) in the number of the immune cells [60,61]. The precise mechanisms by which GNPs bring about this immunosuppressive effect are not clear still now and further studies required in this area [62,63]. As the GNPs are not internalized by lymphocytes, it is possible that their noticeable immunotoxic and cytotoxic effects are mediated by their interactions with cell membranes or related components. It is also possible that the effects observed could be due to the release of ROS-mediated GNPs that caused lipid peroxidation leading to cell and cytoplasmic organelles membrane damage leading to cellular degeneration and necrosis [64]. Overproduction of ROS caused oxidative stress that makes cells unable to maintain the normal physiological functions [65]. The abnormalities in cell function includes proteins and lipid peroxidation [66,67], DNA-strand breaks, modulation of gene expression through activation of transcription factors [68], and modulation of inflammatory responses through signal transduction [69], leading to cell death, immunotoxic and genotoxic effects [70].

There are several oxidative stress markers that reflecting the status of ROS overproduction, including ROS themselves. Nevertheless, ROS are very reactive but have a short half-life. For that reason, it is more applicable to assess oxidative stress via evaluating their oxidation target products, including lipid peroxidation, oxidized proteins, and oxidative nucleic acid damage [71]. In the present study, the group received 15 ppm GNPs showed a significant increase in blood MDA (end product of lipid peroxidation) levels with a significant decrease in CAT activity (antioxidant) accompanied with up-regulation of Nrf-2 mRNA gene levels indicating oxidative stress damage. Since membrane phospholipids are major targets of oxidative damage, lipid peroxidation is often the first parameter analyzed for proving the involvement of free radical damage. Lipid peroxidation produces a progressive loss of cell membrane integrity, impairment in membrane transport function and disruption of cellular ion homeostasis [72]. The increased MDA level following GNPs exposure in our investigation was in agreement with the study of Khan et al. [73] who documented a significant increase in MDA levels in the liver of rats treated with 50 μl of 10 nm-sized GNPs for 3 days. In coincidence with the recorded GNPs-induced significant decrease of blood CAT activity; Shrivastava et al. [74] showed a significant increase in reactive oxygen species (ROS) and depletion of the antioxidant enzyme status in erythrocytes and tissues after 14-day exposure to GNPs. Lingabathula and Yellu [75] recorded a significant increase in the MDA levels with a significant decrease in the GSH levels and CAT activity following exposure of 10 and 25 nm (1 mg/kg bwt) GNPs after 1 day and 1 week from exposures, indicating induction of oxidative stress. About the variation in the oxidant/antioxidant levels between groups received 5 ppm and those received 15 ppm suggesting dose and concentration-dependent oxidative stress induced by GNPs in broilers. The results of histopathological examination in different organs not only reflect the depletion of antioxidants but also reflect the ROS overproduction in group received 15 ppm. However, in 5 ppm GNPs received group showed normal histological structures in all the examined organs with normal oxidant/antioxidants levels as indicating that these particles are generally nontoxic at low doses. Our finding cross linked with other previous study mentioned that GNPs accumulation and cytotoxicity is generally dependent on the dose administered [76].

The oxidative stress can induce DNA damage frequently. The DNA damage includes any change that occurs to the DNA modifying its sequence or changes the major DNA functions [77]. There are many factors that lead to DNA damage such as environmental agents, ROS overproduction, temperature, mutations during DNA replication and methylation [78,79]. The human genome of a non-neuronal cell is exposed to approximately 10,000 lesions that occurred due to endogenous ROS generation. Furthermore, purine base turnover due to the hydrolytic depurination occurs in approximately 2000–10,000 bases in the DNA per day [80]. Our results showed the highest DNA laddering percentage in the hepatic tissue of the group received 15 ppm GNPs (60%) but not observed in 5 ppm received group indicating dose-dependent DNA damage induced by GNPs. This is the first study confirmed that GNPs induced dose-dependent cell and DNA damage. In general, NPs induced cell and DNA damage according to their size, shape, concentration and time of exposure. Rong et al. investigated that there was a dose–response elevation in DNA damage after exposure of A549 cells to different concentration of Nano-Co [81]. Regardless of the pathological alterations observed in the immune organs as discussed previously, there were also remarkable pathological lesions in the liver tissue sections obtained from group received 15 ppm GNPs. The most observable lesions were hepatocellular cytoplasmic vacuolization, necrosis, and hepatic hemorrhage that may be related to GNPs-induced oxidative stress, cell and mitochondrial membrane damage and DNA fragmentation. Several researchers talked about the size and time-dependent DNA damage induced by GNPs but there is no any studies talked about the dose-dependent DNA damage induced by GNPs. Kang et al. [82] showed that no DNA damage was reported in L5178Y cells exposed to 60 nm GNPs while damage occurred with 100 nm GNPs. On the contrary, other studies have shown DNA damage occurred due to exposure to 8 nm GNPs [83] and 20 nm GNPs [84]. The binding affinities between GNPs, amine groups, and thiol stimulate the combinations with biomolecules [85], causing free radical formation due to GNPs exposure [86]. The ultra-small particles are characterized by their large surface areas that can result in the direct formation of ROS that gives rise to cellular damage by damaging the DNA, proteins, and membranes and alter the major functions of mitochondria, cytoplasm and nucleus [87]. Therefore, oxidative stress is a possible mechanism for the induced toxicity of GNPs on DNA and different organs.

## Conclusion

From our findings, we can conclude that the weekly adding of 5 ppm GNPs in drinking water of broiler chickens was effective in increasing the growth performance and immune defense of broilers without affecting the histological structures of the internal organs. On the other hand, the adding of 15 ppm GNPs to drinking water induced extensive cytotoxicity and genotoxicity in broiler chickens as manifested by alterations in the oxidant/antioxidant parameters, histopathological organizations, proinflammatory cytokine levels, and DNA assay. From our results, we recommend adding5ppm GNPs (equivalent to 0.5 mg/ kg), weekly in drinking water of broiler chickens to enhance the growth performance and improve the immune status of broiler chickens.

## Abbreviations

CAT: catalase
FCR: food conversion ratio
GIT: gastrointestinal tract
GNP: gold nanoparticle
LPO: lipid peroxidation
NFE2: nuclear factor erythroid 2
Nrf2: NFE2-related factor 2
POD: peroxidase
ROS: reactive oxygen species
SOD: superoxide dismutase
