# Correction: The effect of different concentrations of gold nanoparticles on growth performance, toxicopathological and immunological parameters of broiler chickens

**DOI:** 10.1042/BSR20194296_COR

**Published:** 2025-03-31

**Keywords:** gene expression, gold nanoparticles, histopathology, inflammation, performance

The authors of the original article titled ‘The effect of different concentrations of gold nanoparticles on growth performance, toxicopathological and immunological parameters of broiler chickens’ (DOI: https://doi.org/10.1042/BSR20194296) would like to correct the affiliation of the author Khaled Yehia Farroh. The original affiliation:

Department of Nanotechnology, Agricultural Research Center, Giza, Egypt

Should instead be: Nanotechnology and Advanced Materials Central Lab., Agricultural Research Center, Giza, Egypt. Regional Center for Food and Feed, Agricultural Research Center, Giza, Egypt.

